# Predicting 7-day, 30-day and 60-day all-cause unplanned readmission: a case study of a Sydney hospital

**DOI:** 10.1186/s12911-017-0580-8

**Published:** 2018-01-04

**Authors:** Yashar Maali, Oscar Perez-Concha, Enrico Coiera, David Roffe, Richard O. Day, Blanca Gallego

**Affiliations:** 1 Centre for Health Informatics, Australian Institute of Health Innovation, Macquarie University, Level 6, 75 Talavera Rd, Sydney, NSW 2109 Australia; 2 Centre for Big Data Research in Health, University of New South Wales, Level 1, AGSM Building (G27), Sydney, NSW 2052 Australia; 3 Chief Information Officer, St Vincent’s Health Australia, Sydney, NSW 2010 Australia; 4 Clinical Pharmacology and Toxicology, St Vincent’s Hospital, Sydney, NSW 2010 Australia; 5 St Vincent’s Clinical School, St Vincent’s Hospital, University of New South Wales, Sydney, Australia

**Keywords:** Hospital readmission, Readmission risk scores

## Abstract

**Background:**

The identification of patients at high risk of unplanned readmission is an important component of discharge planning strategies aimed at preventing unwanted returns to hospital. The aim of this study was to investigate the factors associated with unplanned readmission in a Sydney hospital. We developed and compared validated readmission risk scores using routinely collected hospital data to predict 7-day, 30-day and 60-day all-cause unplanned readmission.

**Methods:**

A combination of gradient boosted tree algorithms for variable selection and logistic regression models was used to build and validate readmission risk scores using medical records from 62,235 live discharges from a metropolitan hospital in Sydney, Australia.

**Results:**

The scores had good calibration and fair discriminative performance with c-statistic of 0.71 for 7-day and for 30-day readmission, and 0.74 for 60-day. Previous history of healthcare utilization, urgency of the index admission, old age, comorbidities related to cancer, psychosis, and drug-abuse, abnormal pathology results at discharge, and being unmarried and a public patient were found to be important predictors in all models. Unplanned readmissions beyond 7 days were more strongly associated with longer hospital stays and older patients with higher number of comorbidities and higher use of acute care in the past year.

**Conclusions:**

This study demonstrates similar predictors and performance to previous risk scores of 30-day unplanned readmission. Shorter-term readmissions may have different causal pathways than 30-day readmission, and may, therefore, require different screening tools and interventions. This study also re-iterates the need to include more informative data elements to ensure the appropriateness of these risk scores in clinical practice.

**Electronic supplementary material:**

The online version of this article (10.1186/s12911-017-0580-8) contains supplementary material, which is available to authorized users.

## Background

Unplanned readmissions to hospital represent a significant burden to health care systems, patients and their families [1]. While not all readmissions can be prevented, there is a consensus that readmission rates across the world are too high and could be reduced through targeted interventions [2–6].

Estimates of how many readmissions are avoidable remain controversial. In the United States all-cause readmissions within 30 days from discharge in 2011 were reported as 15%, and 12% were estimated to be potentially preventable [2, 3]. In the United Kingdom, the emergency 30-day readmission rate between 2004 and 2010 was 7%, and the estimated rate of potentially preventable readmissions was 2% [6]. The Canadian Institute for Health Information reported a rate of 30-day unplanned readmissions of 8.5% [7]. The latest report on returns to acute care in New South Wales, Australia, estimated 16% returns within 30 days after hospitalization for common clinical conditions, and 10% returns within 60 days after common elective surgical procedures [4]. Stroke patients were the most likely to return with a condition deemed to be potentially related to their initial stay, such as a complication or an adverse event (43% of returns). For elective knee replacement, the proportion of returns due to orthopaedic complications was 46% [4].

Many factors can contribute to unplanned readmissions [1, 3, 8–17]. Some are related to deficiencies in quality of care either during the index admission, in the community or in the transition of care. Morbidity and functional disability [10, 12], socioeconomic status [3, 13, 14], and discharge to long-term/nursing facilities [8] have been found to be important general risk factors. Preventable factors under the control of the hospital include management errors, surgical complications, medication related errors, and poor discharge procedures that do not properly involve patients, their relatives, general practitioners or aged-care workers [15, 18, 19]. Some local initiatives to support patients and their caregivers after discharge have been proven to help [16, 17] but wide adoption of sustainable interventions remains elusive. Given limited resources, it makes sense to target those readmissions that hospitals are best able to prevent and to tailor the costliest interventions to patients most likely to benefit from them. This strategy requires methods to accurately, and in a timely manner, estimate risk.

In order to identify the patients that could benefit from discharge planning strategies or other interventions aimed at preventing unwanted returns to hospital, several risk scores have been put forward. We have found six recent (from 2010) existing risk score models of all-cause, 30-day, unplanned [1], emergency, or potentially avoidable readmission: LACE index [20], LACE index + [21], Rothman index [22], HOSPITAL score [23], PARR-30 [24], and PREADM [25]. Typical c-statistic or area under the receiver operating characteristic curve (AUC) for these models ranges from 0.68 (LACE index [20] - prediction includes death -) to 0.75 (LACE index + [21] and Rothman index [22]). Their performance is only fair, when compared, for example, with predictions of mortality using similar data [26, 27]. Part of the problem may lie on the need for additional information, since potential predictors of unplanned readmission span beyond typically available clinical and administrative variables to include patient socio-economic information, patient living arrangements, hospital organisational factors, models of primary and community care available to patients, and patient preferences [28].

In this study, we explored readmission patterns and predictors for all-cause unplanned readmission within 7 days, 30 days and 60 days following discharge from a metropolitan hospital in Sydney, Australia. We utilized routinely available hospital Electronic Health Record (EHR) data together with administrative information on admissions to all other hospitals within the State, which are routinely collected by the Department of Health and linked to the hospital record. We first built a set of predictive models based on a gradient tree boosting algorithm [29]. In the presence of noisy correlated categorical data with unknown interactions, these types of machine learning methods are preferable to the more common logistic regression models [30]. Features selected by these predictive models were then used to develop simple scores, which can be readily used in a hospital setting. Risk scores have less accuracy than their corresponding gradient tree boosting methods but are easy to use in the clinical setting and easy to interpret by users. Patterns and predictors of 7-day versus longer-term readmission were compared.

## Methods

### Settings and study population

Electronic Health Records (EHRs) from 77,776 patients admitted to a 350-bed Sydney teaching, metropolitan hospital between 1 July 2008 and 31 December 2012 were collected. For each patient, an index admission was defined as the first hospital admission by the patient during the study period. Records in the one year before the index admission and 2 months after the index admission from all hospital admissions, emergency department visits and deaths within the State of New South Wales (NSW) were extracted from population health datasets. Namely, the NSW Admitted Patient Data Collection (APDC), the NSW Emergency Department Data Collection (EDDC), and the NSW Registry of Births, Deaths and Marriages (RBDM). The linkage amongst the APDC, EDDC and RBDM was performed by the NSW Centre for Record Linkage using a probabilistic linkage procedure, which guarantees false positive rates <0.5% and false negative rates <0.1% [31]. The linkage between the hospital EHR and the NSW administrative datasets was also carried out independently by the NSW Centre for Record Linkage and only 17 patients could not be linked to the APDC.

**Fig. 1:**
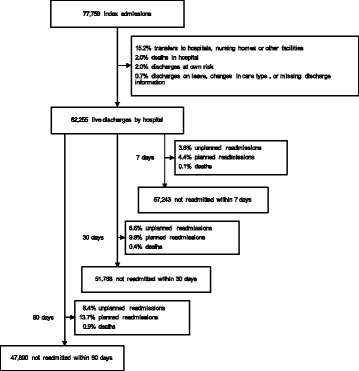
Summary of patients discharged and readmitted over three overlapping periods: 7-days, 30-days and 60-days postdischarge

Of all 77,759 index admissions, 62,255 patients (80.1%) were discharged alive by hospital, 15.2% were followed by transfers to hospitals, nursing homes or other facilities, 2.0% died during admission, 2.0% were discharged at own risk, and the reminder 0.7% represented discharges on leave, changes in type of care or had missing discharge information (Fig. 1).

### Definitions of readmission

A readmission was defined as the first admission to any hospital in New South Wales (NSW) within 60 days of being discharged alive from the index admission. Subsequent readmissions by the same patient or readmissions beyond 60 days were ignored for the purpose of this study. A readmission was defined as unplanned if it was initiated via the emergency department (ED). Amongst the 62,255 patients discharged alive, 13,818 (22.2%) had a readmission within 60 days (5258 of those were unplanned readmissions; 8.4% of the total alive discharges) and 547 died during the follow up period (see Fig. 1). Readmissions were further stratified as occurring within 7 days, 30 days or 60 days from discharge from the index admission.

### Potential predictors

Patients and admissions were characterized using 88 commonly-used variables available in the electronic health record, which can be divided into 5 categories:

1. *Patient demographics*: age, sex, marital status and payment status.
2. *Patient acute-care history*: information on cumulative length of stay (LOS) of hospital admissions within the previous year, as well as time since last admission.
3. *Patient clinical status*: Elixhauser comorbidity groups [32] (defined including one year hospital history), two last common pathology results available before discharge, including hours since last pathology panel. Pathology tests were grouped by their corresponding pathology panels. Results for each panel were classified as missing (if no pathology test within the panel was performed), abnormal (if any test result within the panel was abnormal), or normal.
4. *Admission type*: principal diagnosis, principal procedure type, duration of surgery, type of care, source of referral to hospital, arrival mode and triage code (if coming through ED), ward allocation, LOS, and number of pathology tests and surgeries performed.
5. *Admission and discharge times*: day of the week and time of the day of admission and discharge.

A detailed description of these variables and their distribution in the study population can be found in Additional file 1: Tables S2-S6.

### Pre-processing

Continuous variables were first discretized into categorical variables taking into account domain knowledge and their distribution (Additional file 1: Table S2). Categorical variables were then separated into 211 independent binary variables. The data was separated randomly into two sets: a *derivation set* consisting of 80% of the records and used to derive the final scores and a *validation set* for evaluation.

### Gradient tree boosting models

Separate models were built to predict readmissions within 7 days, 30 days and 60 days from discharge. Each predictive model was built and evaluated using 10-fold cross validation on the derivation set. Patients with a planned readmission were ignored and removed from the derivation and validation datasets. A *gradient tree boosting* [29] algorithm was used for prediction. Gradient tree boosting is a machine learning technique that combines the prediction of an ensemble of weak regression trees, which are added sequentially to the model in order to maximize predictive performance and minimize model complexity. In this study we used the freely available gradient tree boosting algorithm implemented in the R package XGBoost [33] (see Additional file 1: Table S1 for description of model parameters).

### Feature selection

Feature importance was initially quantified using the measure gain provided by XGBoost. Gain represents the improvement in regularized AUC obtained in each split. It is estimated for each feature of each tree and then averaged over all trees. In each cross-validation trial, the top 25 most important features found by the XGBoost algorithm (accounting for over 90% of gain) were selected and included in a logistic regression model. A set of regression parameters was obtained averaging over all cross-validation trials. Statistically significant features (*p*-value < 0.05) for more than 50% of the trials were retained. The mean of the selected features’ distributions for index admissions followed by 7-day readmission, those followed by a readmission between 8 and 30 days and those followed by a readmission between 31 and 60 days were compared using t-test statistics. This comparison was carried out to explore the change of the distribution of important features over different time periods.

### Risk scores

A set of readmission risk scores *RETURN7*, *RETURN 30* and *RETURN 60* were created using the averaged regression parameters of the selected variables. Following Donze et al. [23], scores for each selected variable were assigned by dividing regression parameters by the smallest one and rounding them to the nearest integer.

### Model performance

The discriminative ability of the gradient tree boosting models and the corresponding logistic regression models was estimated via the c-statistic or AUC. We also calculated the sensitivity, specificity and positive predictive value (PPV). For each model, thresholds for these measures were chosen as those that optimized the sum of sensitivity and specificity in the training sets. Both, the average and the standard deviation of these performance measures across all cross-validation trials within the derivation set were reported. The performance of the risk scores was evaluated in the validation set also using AUC, sensitivity, specificity and PPV. Calibration in the validation set was measured via the Hosmer-Lemeshow goodness-of-fit statistics of the observed and expected rate of unplanned readmission across different bins.

## Results

**Fig. 2:**
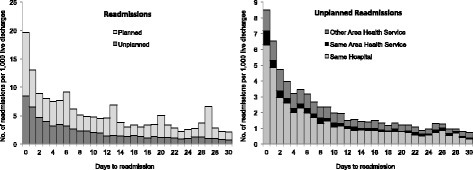
Left panel: Number of planned and unplanned readmissions per 1000 live discharges, per day up to 30 days post-discharge. Right panel: Number of unplanned readmissions per 1000 live discharges, per day up to 30 days post-discharge to the same hospital, other hospitals within the same area health services (AHS) and other hospital in other AHS. Here readmission refers only to the first readmission after discharge. Subsequent readmissions by the same patient have been ignored

Amongst the 62,255 discharges by hospital, 5258 patients (8.4%) returned to hospital via ED within 60 days from discharge, 4101 (6.6%) within 30 days and 2241 (3.6%) within 7 days. The left panel of Fig. 2 shows the rate of planned and unplanned readmissions per day up to 30 days post-discharge. As expected, most readmissions took place shortly after hospitalization. Planned readmissions (hospitalizations not initiated via ED), peaked at weekly intervals post-discharge, reflecting planned weekly returns to hospital. In contrast, unplanned readmissions showed an exponential decrease in the number of readmissions from day of discharge. The majority of unplanned readmissions (65.6%) were assigned an urgent to very urgent ED triage category, requiring treatment within 30 min of presentation. A large percentage of readmissions (37% of unplanned) took place in a different hospital from the index hospital (see right panel in Fig. 2).

The cross-validated predictive performance of the gradient tree boosting models and their corresponding logistic regression models in the derivation set are reported in Table 1. The models achieved a fair performance with AUC for the gradient tree boosting models of 0.71 (for 7-day readmission, which has a very imbalanced dataset where classification categories are very unequally represented), 0.74 (for 30-day readmission) and 0.76 (for 60-day readmission). Table 1 also reports performance measures for the risk scores in the validation set. Risk score discriminative power remained fair, with a small loss of performance compared to the full model. AUC was 0.71 for *RETURN7* and *RETURN30* and 0.74 for *RETURN60*. Sensitivity, specificity and PPV for these scores are reported in Table 1 using the cut-off score that maximised the sum of the sensitivity and specificity in the training sets. Values at additional cut-off points are reported in the Additional file 1: Table S8.

**Table 1:** Models’ discriminative performance

	7-day Readmission			30-day Readmission			60-day Readmission		
	10-fold CV derivation set average (95% CI)		validation set	10-fold CV derivation set average (95% CI)		validation set	10-fold CV derivation set average (95% CI)		validation set
	Gradient Tree Boosting	Logistic Regression (selected variables)	Risk Score: RETURN7	Gradient Tree Boosting	Logistic Regression (selected variables)	Risk Score: RETURN30	Gradient Tree Boosting	Logistic Regression (selected variables)	Risk Score: RETURN60
AUC	0.71 (0.70–0.72)	0.68 (0.67–0.69)	0.71	0.74 (0.73–0.75)	0.72 (0.71–0.73)	0.71	0.76 (0.75–0.76)	0.74 (0.73–0.75)	0.74
SEN (%)	54.7 (52.8–56.6)	71.5 (66.9–76.1)	61.5 (Cut-off = 12)	60.3 (59.0–61.5)	59.0 (56.1–61.9)	52.9 (Cut-off = 12)	63.9 (62.4–65.4)	66.1 (63.0–69.3)	70.0 (Cut-off = 11)
SPE (%)	73.0 (71.9–74.1)	56.5 (51.9–61.0)	69.2 (Cut-off = 12)	73.7 (73.3–74.2)	73.3 (70.7–76.0)	77.4 (Cut-off = 12)	73.4 (72.6–74.2)	69.8 (66.5–73.1)	65.1 (Cut-off = 11)
PPV (%)	7.5 (0.7–0.8)	7.3 (6.7–7.9)	6.9 (Cut-off = 12)	16.0 (15.4–16.5)	16.0 (15.4–16.5)	14.8 (Cut-off = 12)	21.6 (20.9–22.4)	21.4 (20.6–22.2)	18.5 (Cut-off = 11)

*AUC* Area Under the Receiver operating Curve, *SEN* Sensitivity, *SPE* Specificity, *PPV* Positive Predictive Value, *Cut-off* Score cut-off for which SEN, SPE and PPV are calculated

Hosmer-Lemeshow statistics measuring the fit between observed and expected readmission rates showed good calibration for all scores. Observed and expected rates for selected scores can be found in Table 2 and Additional file 1: Table S7.

**Table 2:** Risk Scores’ calibration performance

	7-day Readmission (*β*_0_ = − 4.72, *β*_1_ = 0.14)			30-day Readmission (*β*_0_ = − 4.02, *β*_1_ = 0.13)			60-day Readmission (*β*_0_ = − 3.79, *β*_1_ = 0.13)		
	Number of admissions	Observed readmission rate (%)	Expected readmission rate (%)	Number of admissions	Observed readmission rate (%)	Expected readmission rate (%)	Number of admissions	Observed readmission rate (%)	Expected readmission rate (%)
score = 0	641	0.6	0.9	185	1.1	1.8	135	2.2	2.2
score = 10	1003	3.4	3.4	893	5.8	6.3	707	8.5	7.6
score = 20	98	12.2	12.0	101	21.8	20.4	156	22.4	23.2
score = 30	7	28.6	34.9	10	50.0	49.1	18	55.6	52.5

*Readmission* $Risk\ (S)=\frac{e^{\left({\beta}_0+S\ast {\beta}_1\right)}}{1+{e}^{\left({\beta}_0+S\ast {\beta}_1\right)}}$, where S = score, *β*_0_=intercept, *β*_1_=normalization parameter (full details in Additional file 1: Table S7)

A list of the features used to calculate the scores and their odds ratio (OR) can be found in Table 3. History of hospital admission in the last year, and in particular, cumulative LOS > 7 days (OR equal to 1.79, 2.17 and 3.52 for *RETURN7*, *RETURN30* and *RETURN60* respectively) and previous admission in the last 30 days (OR equal to 1.96, 2.18 and 1.53 for *RETURN7*, *RETURN30* and *RETURN60* respectively) were good predictors of future unplanned readmission in all risk scores. Old age and whether the index admission was an emergency admission was also important (see Table 3). Amongst the comorbidity groups, solid tumor without metastasis, psychosis and drug abuse were the prevalent predictors in all readmission groups. An abnormal test result before discharge as part of the frequently performed pathology panels: full blood count (FBC), Urea, Electrolytes and Creatinine (UEC), or Liver Function Tests (LFT), was also a predictor of readmission. In two situations (lipase for 7-day readmission and INR for 60-day readmission), in which most of the test results were either normal or missing, normal results versus no tests were associated with unplanned readmission. The two socio-economic variables (marital status and payment status) that were available in the medical record appeared important, as unmarried public patients were more likely to be readmitted. Overseas visitors had less probability of unplanned readmission at 60-days, probably reflecting lack of follow-up.

**Table 3:** Risk scores for all-cause unplanned readmission (in bold common predictors to all risk scores; in italic predictors with negative associated scores)

7-days readmission			30-days readmission			60-days readmission		
Feature	Score	OR (95%CI)	Feature	Score	OR (95%CI)	Feature	Score	OR (95%CI)
**Last admission in ≤ 1 month**	5	1.96 (1.65–2.30)	**Last admission in ≤ 1 month**	6	2.18 (1.88–2.50)	**CumLOS in past year > 7 days**	10	3.52 (3.13–3.93)
**Admitted via ED**	4	1.81 (1.52–2.12)	**CumLOS in past year > 7 days**	6	2.17 (1.83–2.54)	**Older than 85**	5	1.88 (1.62–2.18)
**CumLOS in past year > 7 days**	4	1.79 (1.49–2.13)	**Solid tumor without metastasis**	5	1.87 (1.63–2.14)	0 < CumLOS in past year ≤ 7 days	5	1.83 (1.72–1.96)
**Solid tumor without metastasis**	4	1.71 (1.48–2.00)	**Admitted via ED**	4	1.71 (1.55–1.89)	**Solid tumor without metastasis**	4	1.70 (1.30–2.17)
**Psychosis**	4	1.67 (1.21–2.24)	**Older than 85**	3	1.55 (1.29–1.85)	**Psychosis**	4	1.69 (1.44–1.97)
*Last surgery in ≤ 6 h*	*-3*	*0.65 (0.60–0.71)*	**Psychosis**	3	1.49 (1.08–1.96)	Metastatic cancer	4	1.62 (1.18–2.14)
**Older than 85**	3	1.49 (1.20–1.84)	**Drug abuse**	3	1.46 (1.14–1.82)	**Admitted via ED**	3	1.57 (1.44–1.71)
Rheumatoid arthritis	2	1.37 (1.17–1.60)	Diabetes, complicated	3	1.40 (1.17–1.65)	**Last admission in ≤ 1 month**	3	1.53 (1.23–1.85)
Alcohol abuse	2	1.35 (1.08–1.67)	**Medicare public patient**	2	1.37 (1.27–1.47)	**Drug abuse**	3	1.46 (1.15–1.80)
**Medicare public patient**	2	1.34 (1.21–1.49)	Cardiac arrhythmia	2	1.32 (1.13–1.51)	Congestive heart failure	3	1.41 (1.05–1.82)
Depression	2	1.32 (1.04–1.64)	Chronic pulmonary disease	2	1.31 (1.10–1.54)	**Medicare public patient**	3	1.41 (1.31–1.51)
**Drug abuse**	2	1.31 (0.94–1.74)	Abnormal last CRP	2	1.27 (1.14–1.42)	Alcohol abuse	2	1.36 (1.15–1.58)
Normal last Lipase	2	1.29 (1.10–1.50)	Allied Health Intervention	2	1.26 (1.09–1.45)	*Overseas visitor*	*-2*	*0.76 (0.70–0.83)*
**Abnormal last FBC**	2	1.28 (1.05–1.53)	**Abnormal last FBC**	1	1.20 (1.02–1.39)	Age between 65 and 85	2	1.30 (1.20–1.40)
Abnormal last CRP	1	1.23 (1.06–1.41)	Normal last Blood Alcohol	1	1.19 (1.04–1.35)	Allied Health Intervention	2	1.22 (1.06–1.39)
Last ward = Emergency/Mobile Unit	1	1.22 (1.08–1.37)	**Abnormal last LFT**	1	1.18 (1.04–1.34)	**Abnormal last FBC**	1	1.21 (1.05–1.38)
**Not Married**	1	1.21 (1.08–1.36)	**Abnormal last UEC**	1	1.17 (1.04–1.32)	**Abnormal last LFT**	1	1.21 (1.08–1.35)
Discharged between 14:00–24:00	1	1.18 (1.07–1.29)	LOS > 7 days	1	1.15 (0.98–1.35)	Normal last INR	1	1.21 (1.07–1.36)
**Abnormal last UEC**	1	1.17 (1.01–1.35)	**Not Married**	1	1.14 (1.05–1.25)	*Referred by other practitioner*	*−1*	*0.83 (0.80–0.87)*
**Abnormal last LFT**	1	1.15 (0.98–1.33)				**Not Married**	1	1.18 (1.09–1.27)
						**Abnormal last UEC**	1	1.16 (1.04–1.29)
						LOS > 7 days	1	1.15 (0.97–1.33)
						Number of panels > 10	1	1.14 (1.01–1.27)

**Fig. 3:**
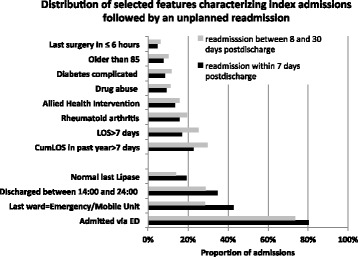
Distribution of selected features characterizing index admissions that are followed by unplanned readmission within 7 days from discharge or unplanned readmission between 8 and 30 days from discharge. Selected features are those for which the difference in proportions is statistically significant. LOS = Length of Stay; CumLOS=Cummulative LOS; ED = Emergency Department; Emergency/Mobile ward refers to Emergency ward or mobile acute treatment units

Some features, such as time since last surgery, last ward or discharge time, which were important for predicting 7-day readmission, were less important for predicting longer-term readmission. Conversely, variables such as LOS, cumulative LOS (cumLOS) in the past year, age over 85 and allied health intervention were better predictors of 30-day and 60-day readmission. Figure 3 shows the distributions of risk scores features across index admissions followed by 7-day unplanned readmission that were statistically different from the means features’ distributions across index admissions followed by 8 to 30-day unplanned readmission. Unplanned readmission after the first week post-discharge was associated with longer index admission (25% had LOS > 7 days, compared to 17% for 7-day readmission). They were also associated with sicker patients with average number of comorbidity groups 2 (versus 1 for 7-day readmission) and higher use of acute care in the past year. Differences in feature distributions between the 8–30 day and 31–60 day readmission groups were less significant.

## Discussion

The aim of this study was to investigate the factors associated with unplanned readmission in a Sydney hospital. We started by measuring the number of unplanned readmissions per day to the same hospital, as well as to other hospitals within the State. We then developed and compared validated readmission risk scores using routinely collected hospital data to predict 7-day (*RETURN7*), 30-day *(RETURN30)* and 60-day (*RETURN60)* all-cause unplanned readmission. The AUC was 0.71 for *RETURN7* and *RETURN30* and 0.74 for *RETURN60.*

Given that hospitals struggle to keep up with growing demands from a rising number of hospitalizations, and that not all readmissions can be prevented, it is important to target interventions to patients that are most likely to benefit from them. Some strategies that have proved to be beneficial are costly and require additional qualified staff. As a result, they have not been widely adopted [34]. In this scenario, risk scores are an appropriate and easy-to-implement tool that can help identify high-risk patients before discharge. This has the potential to help target those for whom readmissions can be avoided for example with special transitional care, delayed discharge or provision of alternative care.

In this study, we found that a significant number of unplanned readmissions took place in hospitals different from the hospital of the index admission (see right panel of Fig. 2). This was confirmed in the latest report on readmissions in New South Wales [4]. It reflects the importance of maintaining medical record systems that are patient (as opposed to visit) centric, and can follow the patient across institutions [35]. It also has implications for the implementation of financial penalties for unplanned returns to hospital.

When compared to existing models of 30-day unplanned readmission, our model confirms much of what has already been observed in previous work [20–25]. Our predictive power is similar to that of the best available published models. Our choice of threshold to stratify patients into two groups (no unplanned readmission and unplanned readmission) was built to optimize the combined sum of sensitivity or recall and specificity. An alternative threshold could have been chosen to optimize PPV. For example, a higher cut-off score of 20 points in RETURN30 (see Additional file 1: Table S8) provided PPV = 28% but a Sensitivity = 18%. Other scores like e.g. PARR-30 [24] reported a PPV = 59% and Sensitivity = 5.4% for scores above 50%. A high sensitivity ensures that most patients at high risk of readmission are correctly identified. On the other hand, a high PPV could contain the costs of readmission strategies since it ensures patients selected for an intervention are likely to benefit from it.

In this study we make use of a modelling technique known as *gradient tree boosting* [29]; uncommon in the construction of previous readmission models, but popular in the machine learning community. Although performing variable selection with this algorithm does not appear to provide improved predictive performance when compared to previous work, existing high-performing models were trained in much larger datasets. Further work is needed to assess if performance here could improve with larger sample sizes.

In addition, due to the fact that outcome categories are very unequally represented, with a much larger number of no readmissions than unplanned readmissions, it was easier to predict unplanned readmissions to hospital within 60 days post-discharge (AUC = 0.74) than within 7 days (AUC = 0.71).

A patient’s history of health care utilization in the previous year was found to be the most important predictor of unplanned readmission in all models. This agrees with the previous literature which found number of hospital admissions [21, 23–25], number of emergency department visits [20, 21], and number of primary care and specialist visits [25] in the past year to be important predictors. Number of previous hospital admissions was strongly correlated with cumulative LOS across these admissions. The latter was chosen as the better proxy for acute care utilization. Similarly, the number of days since last admission was a common predictor found in previous work [24, 25], as was urgency of the index admission [20, 21, 23]. Unmarried patients and public (Medicare-holder) patients were more likely to have an unplanned readmission. Australia’s publicly funded health care system entitles citizens and most permanent residents to be eligible for Medicare. Medicare services include treatment in public hospitals, subsidised treatment in private hospitals, subsidised outpatient services and subsidised access to medicines prescribed in private hospitals and the community.

Abnormal results for commonly performed pathology tests (in particular within the FBC, UEC and LFT panels) before discharge were also found to be important features. This agrees with Donze et al. [23], who found low haemoglobin and low sodium at discharge to be predictive of potentially avoidable readmissions. Another similarity with Donze et al., is the identification of a cancer diagnosis as a predictor of unplanned readmission. Several previous studies have found high rates of unplanned readmissions for cancer patients [36–38]. Analysis of risk factors for these patients pointed at severity of illness and procedure complications as reasons for these high rates. These findings raise the issue of improving oncology care in primary and community care. Potentially preventable hospitalizations for very ill cancer patients and the need to improve access to palliative care outside hospitals has also been discussed in the context of the ‘weekend effect’ [39] (difference in mortality observed in patients admitted to hospital during the weekend versus weekdays).

We found that some predictors of readmission within 30 and 60 days post-discharge (such as LOS and allied health intervention) were not relevant for shorter-term predictions. Conversely, time since last surgery, last ward and discharge time were predictors of unplanned readmission within 7 days post-discharge but did not affect the prediction of longer-term readmissions. Analysis of predictors’ distributions in the group that had a 7-day readmission versus 8 to 30-day readmission confirmed that longer-term readmissions where more frequently associated with older patients, longer hospital stays, higher use of acute care in the past year and more comorbidities; while short-term readmissions were more frequently associated with urgent admissions. This is an indication that some shorter-term readmissions may have different causality than longer-term readmissions.

### Limitations and future work

In this study, a readmission is considered ‘unplanned’ if it takes place via the Emergency Department. By using this definition, we may be missing unplanned admissions to hospital initiated by specialists, who directly admit their patients into hospital prompted by unexpected events. Furthermore, an unplanned readmission does not necessarily imply preventability. A modification of our models considering validated definitions of potentially preventable readmission is left as future work. Although several definitions of potentially preventable readmissions have been put forward [40, 41], current readmission models have not been compared using the same definition. Standardising this concept would lead to appropriate comparisons across predictive techniques and their corresponding scores. More importantly, current scores only have fair discrimination ability. Inclusion of more informative data elements should be taken into account if we are to use these scores in clinical practice.

The robustness of the risk scores, particularly regarding the weights of the less important predictors can be improved with larger training datasets. This is particularly the case in the 7-day readmission model, where less than 2000 unplanned readmissions are available in the derivation set. Also, the effect of discretising continuous variables was not explored in this study and may have influenced the prediction performance of the scores. This study did not include any investigation to establish causality between predictors and unplanned readmission. Furthermore, this study is limited to index admissions to a 350-bed teaching, metropolitan hospital in Sydney. Therefore, population characteristics reflect those of the catchment area of this hospital. The inclusion of larger sample sizes, investigation of causality for selected predictors and external validation using records from different hospitals can produce more robust and clinically meaningful scores. This has been left for future work.

## Conclusions

This study developed risk scores to identify 7-day, 30-day and 60-day all-cause unplanned readmission in a Sydney hospital. The models achieved a fair predictive performance, similar to current models trained with larger datasets. Additional variables not currently contained in EHR data may be needed to improve performance. There is some indication that 7-day unplanned readmissions may have different causal pathways than longer-term readmissions. Overall, it may be more beneficial to design screening tools that identify candidates for appropriate preventive interventions, such as candidates that may benefit from delayed discharge, or candidates that should be offered alternative care pathways.

## Additional file

Additional file 1: Table S1. Parameters of the Gradient Tree Boosting algorithm. In this study, we used the freely available gradient tree boosting algorithm implemented in the R package XGBoost with the following parameters chosen via manual tuning. **Table S2.** Conversion of continuous variables into categorical variables: cutting points for hospital length of stay (LOS), age (years), cumulative LOS (hours) in the previous year, days from last admission, number of pathology tests, number of pathology panels, hours since last surgery, hours since last panel and admission type. **Table S3**: Characteristics of patients and their hospital admissions for the study population. Main descriptive statistics. **Table S4.** Main categories of primary diagnosis (ICD10-AM) in our cohort. **Table S5.** Comorbidity groups in our cohort (Reference value = no comorbidity). **Table S6.** Pathology variables identified by the hospital laboratory in our cohort (Reference value = missing). **Table S7.** Calibration performance; Observed and expected rates for selected scores can be found in this table. **Table S8.** Sensitivity, specificity and PPV for different cut-off scores. (PDF 155 kb)

## Abbreviations

APDC: Admitted Patient Data Collection
AUC: Area under the receiver operating characteristic curve
CRP: C-Reactive Protein
CumLOS: Cumulative length of stay in the last year from index admission
ED: Emergency Department
EDDC: Emergency Department Data Collection
EHR: Electronic Health Record
FBC: Full blood count
HOSPITAL: “H” stands for hemoglobin at discharge; “O” discharge from an oncology service; “S” sodium level at discharge; “P” procedure during the index admission; “I” index “T” type of admission; “A” number of admissions during the last 12 months; “L” length of stay
INR: International normalized ratio
LACE: “L” stands for length of stay of the index admission; “A”: acuity of the admission; “C”: comorbidity; “E”: Emergency Department visits in the last 6 months
LFT: Liver Function Tests
LOS: Length of stay
NSW: New South Wales
PARR − 30: Patients at risk of readmission within 30 days
PPV: Positive predictive value
PREADM: Preadmission readmission detection model
RBDM: Registry of Births, Deaths and Marriages
UEC: Urea, Electrolytes and Creatinine
XGBoost: eXtrem Gradient Boosting
