# Supplementary material for: Predicting 7-day, 30-day and 60-day all-cause unplanned readmission: a case study of a Sydney hospital
Source: BMC Med Inform Decis Mak. 2018 Jan 4;18:1. doi: 10.1186/s12911-017-0580-8 (PMC5755362; doi:10.1186/s12911-017-0580-8)
Supplement: Additional file 1: Table S1. — Parameters of the Gradient Tree Boosting algorithm. In this study, we used the freely available gradient tree boosting algorithm implemented in the R package XGBoost with the following parameters chosen via manual tuning. Table S2. Conversion of continuous variables into categorical variables: cutting points for hospital length of stay (LOS), age (years), cumulative LOS (hours) in the previous year, days from last admission, number of pathology tests, number of pathology panels, hours since last surgery, hours since last panel and admission type. Table S3: Characteristics of patients and their hospital admissions for the study population. Main descriptive statistics. Table S4. Main categories of primary diagnosis (ICD10-AM) in our cohort. Table S5. Comorbidity groups in our cohort (Reference value = no comorbidity). Table S6. Pathology variables identified by the hospital laboratory in our cohort (Reference value = missing). Table S7. Calibration performance; Observed and expected rates for selected scores can be found in this table. Table S8. Sensitivity, specificity and PPV for different cut-off scores. (PDF 155 kb) [file 12911_2017_580_MOESM1_ESM.pdf]

Supplementary Material

Table S1: Parameters of the Gradient Tree Boosting algorithm. In this study, we used the freely available gradient tree boosting algorithm implemented in the R package XGBoost with the following parameters chosen via manual tuning:		
**Parameter**	**Value**	**Variable name in R package XGBoost [23]**
Number rounds	1,000	nrounds
Maximum depth of each tree	5	max_depth
Factor scaling the contribution of each tree	0.01	eta
Minimum loss reduction required to make a further partition on a leaf node of the tree	1	gamma
Maximum gradient delta step for tree weight estimation	1	max_delta_step
Minimum sum of instance weigh in a child	5	min_child_weight
Subsample ratio of the training instance	0.5	subsample
Subsample ratio of columns when constructing each tree	0.6	colsample_bytree

Table S2: Conversion of continuous variables into categorical variables (cutting points)		
**Name of the feature**	**Number of levels**	**Cutting points**
LOS (hours)	6	12, 24, 48, 72, 168
Age (years)	5	25, 45, 65, 85
Cumulative LOS (hours)	3	0, 168
Days from last admission	3	30, 365
Number of pathology tests	6	0, 25, 50, 100, 200
Number of pathology panels	5	0, 1, 5, 10
Hours since last surgery	4	(no surgery), 6, 48
Hours since last panel	5	(no panel), 0, 6, 24
Admission type	3	(Medical), Minor Surgery*, Surgery**

*: Minor Surgery is defined as surgeries with not more than 1 hour duration.
**: Surgery is defined as surgeries with more than 1 hour duration.

Table S3: Characteristics of patients and their hospital admissions for the study population

Feature	25th-Perc.	Median	Mean	75th-Perc.	STD	Reference value
**Age (years)**	34.0	52.0	51.9	68.0	20.1	<=25 years
**Cumulative LOS in the past year (hours)**	0	0	85.1	5.9	343.5	0 hours
**Days since last admission**	237.8	365.5	290.2	365.5	127.5	>365 days (no admission)
**Number of pathology tests**	3.0	37.0	83.5	70.0	226.0	0
**Number of pathology panels**	2.0	8.0	10.1	13.0	11.1	0
**Hours since last surgery**	0	0	22.4	5.0	74.6	(no surgery)
**Length of stay (hours)**	6.8	22.6	74.3	74.4	166.6	<=12 hours

Feature	Category	Statistics	Reference value
**Gender (%)**	Male	57	Male
	Female	43	
**Day of Week Admission (%)**	Mon	17.1	Mon
	Tue	17.1	
	Wed	16.3	
	Thu	17.3	
	Fri	14.1	
	Sat	9.0	
	Sun	8.8	
**Day of Week Discharge (%)**	Mon	15.5	Mon
	Tue	17.0	
	Wed	16.7	
	Thu	17.6	
	Fri	16.7	
	Sat	9.1	
	Sun	7.1	
**Time of Day Admission (%)**	00:00-07:59	25.9	08:00-15:59
	08:00-15:59	47.4	
	16:00-23:59	26.5	
**Time of Day Discharge (%)**	00:00-07:59	4.9	08:00-15:59
	08:00-15:59	64.1	
	16:00-23:59	30.9	
**Marital Code (%)**	Married	39.3	Married
	Never Married	40.2	
	Widow	6.8	
	Divorced/separated	6.3	
	Unknown	7.2	
**Episode of care type (%)**	Acute care	99.2	Acute care
	Palliative care	0.4	
	Rehabilitation care	0.2	
**Source of referral (%)**	Emergency Department	61.7	Others
	Outpatients	9.3	
	Other Hospital	1.8	
	Other Medical Practitioner other than Private Psychiatric Practice	24.6	
	Hospital in same AHS	0.4	
	Private Psychiatric Practice	0.0	
	Others	2.1	
**Admission type**	Medical	61.2	Medical
	Minor Surgery	19.6	
	Surgery	19.2	
**Triage Code (%)**	Resuscitation	3.5	Ref=No ED
	Emergency	13.8	
	Urgent	35.0	
	Semi-urgent	8.5	
	Non-urgent	0.3	
	No ED	38.7	
**Arrival mode (%)**	Community/Public Transport	17.7	Not from ED
	Police/Correctional Services Vehicle	1.1	
	Private Car	12.7	
	Ambulance Service	29.5	
	Air Ambulance	0.0	
	Not from ED	38.7	
	Unknown	0.1	
**Payment status (%)**	Medicare card holder and public patient	69.1	other or missing
	Medicare card holder and private patient	18.3	
	Overseas visitor	7.6	
	Veterans affairs	1.5	
	Prisoner	0.0	
	Trial patient	0.0	
	Other or missing	3.3	

**Ward type**	Emergency/Mobile Unit	33.7	Others
	Day Procedure	19.0	
	Cardiology	8.2	
	Cardiothoracic Surgery and Transplant	6.1	
	Orthopaedics, Urology, Rheumatology and Plastics	5.7	
	Neurosurgery, neurology, vascular medicine, vascular surgery, stroke unit	5.6	
	Oncology, haematology, bone marrow transplant, immunology, nephrology, rheumatology, HIV, clinical pharmacology, drugs & alcohol	5.1	
	Psychiatric emergency care centre	2.3	
	Gastroenterology, Colorectal surgery, GIT	6.9	
	Mental Health	1.8	
	ICU	1.1	
	Geriatrics	2.5	
	Others	1.9	
**Procedure blocks**	Allied Health Intervention	9.1	No procedure
	Haemodialysis	4.9	
	Computerised tomography of brain	4.8	
	Coronary angiography	3.0	
	Panendoscopy with excision	2.3	
	Computerised tomography of abdomen and pelvis	1.7	
	Administration of blood and blood products	1.6	
	No procedure	27.1	
	Others	45.1	

**Table S4:** Categories of primary diagnosis in our cohort, ICD10			
Cardiac murmurs and other cardiac sounds	4.21%	Gastritis and duodenitis	1.31%
Abdominal and pelvic pain	2.30%	Other forms of heart disease	1.29%
Ischaemic heart diseases (I20)	2.23%	Mental and behavioural disorders due to use of alcohol	1.27%
Syncope and collapse	1.86%	Acute appendicitis	1.17%
Transient cerebral ischaemic attacks and related syndromes	1.55%	"Others"	81.4%
Ischaemic heart diseases (I25)	1.35%		

**Table S5:** Comorbidity groups in our cohort (Reference value= no comorbidity)			
**Comorbidity groups**	**Percentages**	**Comorbidity groups**	**Percentages**
Congestive heart failure (%)	3.6	AIDS/HIV (%)	1.2
Cardiac arrhythmia (%)	10.1	Lymphoma (%)	0.8
Valvular disease (%)	2.6	Metastatic cancer (%)	2.3
Pulmonary circulation disorders (%)	1.3	Solid tumor without metastasis (%)	5.1
Peripheral vascular disorders (%)	2.0	Rheumatoid arthritis/collagen (%)	1.4
Hypertension Uncomplicated (%)	21.0	Coagulopathy (%)	1.2
Hypertension Complicated (%)	0.1	Obesity (%)	2.0
Paralysis (%)	0.6	Weight loss (%)	0.0
Other neurological disorders (%)	3.3	Fluid and electrolyte disorders (%)	5.3
Chronic pulmonary disease (%)	6.5	Blood loss anemia (%)	0.0
Diabetes uncomplicated (%)	2.9	Deficiency anemia (%)	1.5
Diabetes complicated (%)	5.8	Alcohol abuse (%)	7.2
Hypothyroidism (%)	1.7	Drug abuse (%)	4.3
Renal failure (%)	3.4	Psychoses (%)	2.8
Liver disease (%)	2.0	Depression (%)	6.6
Peptic ulcer disease excluding bleeding (%)	0.7		

**Table S6:** Pathology variables identified by the hospital laboratory in our cohort (Reference value=missing)									
Panel	Pathology tests	Normal (%)	Abnormal (%)	Missed (%)	Panel	Pathology tests	Normal (%)	Abnormal (%)	Missed (%)
Panel 1: Full Blood count (FBC)	Haemoglobin<br>White Blood Cell<br>RBC<br>Red Cell Distribution Width<br>Mean Corpuscular Hemoglobin Concentration<br>Hematocrit test<br>Mean Corpuscular Volume<br>Mean Corpuscular Hemoglobin<br>Platelets<br>Meosinophils<br>Monocytes<br>Basophils<br>Lymphocytes<br>Neutrophils	11.5	58.9	29.5	Panel9: International Normalised Ratio (INR)	International Normalised Ratio	55.2	15.1	29.7
Panel2: Urea Electrolyte and creatinine (UEC)	Creatinine<br>Urea<br>Sodium<br>Potassium<br>Chloride<br>Bicarbonate<br>Estimate Glomerular Filtration Rate	31.7	38.6	29.7	Panel10: Troponin I	Troponin I	61.9	8.4	29.7
Panel3:Liver Function Test (LFT)	Albumin<br>Total Protein<br>Aspartate Aminotransferase<br>Gamma Glutamyl Transferase<br>Alanine Aminotransferase<br>Total Bilirubin<br>Alkaline Phosphatase	22.3	38.9	38.6	Panel11: Lipase	Lipase	65.5	4.8	29.7
Panel4:CA/MG/PHOS	Calcium<br>Magnesium<br>Inorganic Phosphate	38.6	14.1	47.2	Panel12: Transferrin saturation	Transferrin saturation	67.7	2.5	29.7
Panel5: C-Reactive Protein (CRP)	C-Reactive Protein	69.5	0.9	29.5	Panel13: Faecal Occult Blood	Faecal Occult Blood	52.0	18.2	29.7
Panel6: Activated Partial Thromboplastin Time (APTT)	Activated Partial Thromboplastin Time	42.2	28.3	29.5	Panel14: Blood Alcohol Concentration	Blood Alcohol Concentration	59.0	10.7	30.3
Panel7: Glucose	Glucose	52.2	18.2	29.5	Panel15: GPScreen	GPScreen	53.6	7.7	38.7
Panel8: Prothrombin Time (PT)	Prothrombin Time	61.0	9.3	29.7	Panel16: Free thyroxine	Free thyroxine	51.1	9.6	38.9

Table S7: Calibration performance; Observed and expected rates for selected scores can be found in this table.

7-days				30-days				60-days			
S*	Number Admission	Readmission rate		S*	Number Admission	Readmission rate		S*	Number Admission	Readmission rate	
		Observed	Expected			Observed	Expected			Observed	Expected
-3	115	0.0%	0.6%	0	185	1.1%	1.8%	-3	2	0.0%	1.5%
-2	144	0.7%	0.7%	1	187	1.6%	2.0%	-2	9	0.0%	1.7%
-1	366	0.0%	0.8%	2	693	1.4%	2.3%	-1	116	0.9%	1.9%
0	641	0.6%	0.9%	3	1049	2.3%	2.6%	0	135	2.2%	2.2%
1	299	0.7%	1.0%	4	346	3.5%	3.0%	1	85	1.2%	2.5%
2	383	0.8%	1.2%	5	555	2.9%	3.4%	2	458	1.7%	2.8%
3	524	0.8%	1.3%	6	657	3.7%	3.8%	3	867	2.7%	3.2%
4	322	2.2%	1.5%	7	1011	4.6%	4.3%	4	684	3.4%	3.6%
5	381	0.5%	1.7%	8	1084	5.4%	4.9%	5	592	3.9%	4.1%
6	470	1.3%	2.0%	9	1072	6.3%	5.6%	6	534	4.1%	4.7%
7	636	3.1%	2.3%	10	893	5.8%	6.3%	7	709	6.6%	5.3%
8	803	1.6%	2.6%	11	707	6.6%	7.2%	8	801	6.1%	6.0%
9	947	3.5%	3.0%	12	552	9.1%	8.1%	9	893	7.4%	6.8%
10	1003	3.4%	3.4%	13	432	9.7%	9.2%	10	707	8.5%	7.6%
11	1069	3.4%	3.8%	14	355	14.6%	10.3%	11	584	10.3%	8.6%
12	929	5.3%	4.4%	15	300	14.0%	11.6%	12	471	11.3%	9.7%
13	733	6.0%	5.0%	16	220	9.5%	13.1%	13	446	12.1%	10.8%
14	565	5.3%	5.7%	17	157	16.6%	14.6%	14	421	15.7%	12.2%
15	424	5.7%	6.5%	18	150	16.0%	16.4%	15	338	16.0%	13.6%
16	277	5.8%	7.3%	19	116	12.9%	18.3%	16	281	16.4%	15.2%
17	204	6.9%	8.3%	20	101	21.8%	20.4%	17	257	19.8%	17.0%
18	159	7.5%	9.4%	21	80	26.3%	22.6%	18	195	17.9%	18.9%
19	141	8.5%	10.7%	22	70	28.6%	25.0%	19	178	20.8%	20.9%
20	98	12.2%	12.0%	23	61	23.0%	27.6%	20	156	22.4%	23.2%
21	73	20.5%	13.6%	24	41	29.3%	30.3%	21	121	24.8%	25.6%
22	49	12.2%	15.2%	25	44	34.1%	33.2%	22	124	29.0%	28.1%
23	44	27.3%	17.1%	26	28	32.1%	36.2%	23	106	27.4%	30.8%
24	34	14.7%	19.1%	27	17	29.4%	39.3%	24	87	32.2%	33.6%
25	20	10.0%	21.3%	28	13	46.2%	42.5%	25	67	35.8%	36.6%
26	19	26.3%	23.7%	29	14	35.7%	45.8%	26	70	37.1%	39.6%
27	13	23.1%	26.3%	30	10	50.0%	49.1%	27	59	37.3%	42.8%
28	2	0.0%	29.0%	31	5	40.0%	52.5%	28	53	47.2%	46.0%
29	5	20.0%	31.9%	32	1	0.0%	55.8%	29	39	46.2%	49.2%
30	7	28.6%	34.9%	34	3	33.3%	62.2%	30	18	55.6%	52.5%
31	5	0.0%	38.1%	35	1	0.0%	65.3%	31	16	56.3%	55.7%
34	1	0.0%	48.1%					32	10	50.0%	58.9%
								33	6	50.0%	62.0%
								34	6	16.7%	65.0%
								35	1	0.0%	67.9%
								36	1	100.0%	70.6%
								40	1	100.0%	80.2%

S*=Score

Table S8: Sensitivity, specificity and PPV for different cut-off scores

RETURN7					***RETURN30***					***RETURN60***				
**cut-off score**	10	12	20	30	**cut-off score**	10	12	20	30	**cut-off score**	10	11	20	30
**SEN**	77.9%	61.5%	14.7%	0.5%	**SEN**	65.7%	52.9%	17.7%	1.0%	**SEN**	75.5%	70.0%	27.9%	2.8%
**SPE**	51.7%	69.2%	97.3%	99.9%	**SPE**	63.0%	77.4%	96.6%	99.9%	**SPE**	58.4%	65.1%	93.4%	99.7%
**PPV**	5.7%	6.9%	17.0%	15.4%	**PPV**	11.6%	14.8%	28.0%	40.0%	**PPV**	17.0%	18.5%	32.2%	50.8%
